# Small Molecule Metabolite Biomarker Candidates in Urine from Mice Exposed to Formaldehyde

**DOI:** 10.3390/ijms150916458

**Published:** 2014-09-17

**Authors:** Juan Zhang, Rongli Sun, Yue Chen, Kehong Tan, Haiyan Wei, Lihong Yin, Yuepu Pu

**Affiliations:** Key Laboratory of Environmental Medicine Engineering, Ministry of Education, School of Public Health, Southeast University, Nanjing 210009, China; E-Mails: sunrongli5318@gmail.com (R.S.); chenyue_0531@126.com (Y.C.); kehom123@126.com (K.T.); why_314614@163.com (H.W.); lhyin@seu.edu.cn (L.Y.)

**Keywords:** formaldehyde, biomarker, metabonomics, urine, HPLC-TOF-MS

## Abstract

Formaldehyde (FA) is a ubiquitous compound used in a wide variety of industries, and is also a major indoor pollutant emitted from building materials, furniture, *etc.* Because FA is rapidly metabolized and endogenous to many materials, specific biomarkers for exposure have not been identified. In this study, we identified small metabolite biomarkers in urine that might be related FA exposure. Mice were allowed to inhale FA (0, 4, 8 mg/m^3^) 6 h per day for 7 consecutive days, and urine samples were collected on the 7th day of exposure. Liquid chromatography coupled with time of flight-mass spectrometry and principal component analysis (PCA) was applied to determine alterations of endogenous metabolites in urine. Additionally, immune toxicity studies were conducted to ensure that any resultant toxic effects could be attributed to inhalation of FA. The results showed a significant decrease in the relative rates of T lymphocyte production in the spleen and thymus of mice exposed to FA. Additionally, decreased superoxide dismutase activity and increased reactive oxygen species levels were found in the isolated spleen cells of exposed mice. A total of 12 small molecules were found to be altered in the urine, and PCA analysis showed that urine from the control and FA exposed groups could be distinguished from each other based on the altered molecules. Hippuric acid and cinnamoylglycine were identified in urine using exact mass and fragment ions. Our results suggest that the pattern of metabolites found in urine is significantly changed following FA inhalation, and hippuric acid and cinnamoylglycine might represent potential biomarker candidates for FA exposure.

## 1. Introduction

Formaldehyde (FA) is a ubiquitous organic compound that is colorless, flammable and has a distinctive strong odor [1]. Exposure to FA occurs in a wide variety of occupations and industries, including the construction, textile, paper product, resin, wood composite, insulating material, paint, plastic, fabric, adhesive, and cosmetic industries [2,3]. FA is also a major indoor air pollutant emitted from building materials, furniture, chipboards, fabrics, as well as in heating and cooking fumes [4,5]. Additionally, FA is an endogenous metabolite and essential metabolic intermediate in all cells.

FA has been recognized as a carcinogen and strong mutagenic agent by the International Agency for Research on Cancer, and several studies have shown that FA induces leukemia and formation of squamous-cell carcinomas in the nasal mucosa [6].While FA predominantly causes allergic reactions such as contact dermatitis and occupational asthma, it can also produce adverse effects on immunity, which may alter lymphocyte subpopulations and cytokine levels in exposed individuals [7,8]. Because FA is both rapidly metabolized and endogenously present [9,10], inhalation of FA does not seem to lead to its increased concentration in blood and, therefore, specific biomarkers for FA exposure have not been identified. It has been reported that FA concentrations in blood samples obtained from volunteers who inhaled 1.9 ppm FA for 40 min did not differ from pre-exposure concentrations [11]. To differentiate between exogenous and endogenous FA, rats were exposed to stable isotope (13C)-labeled FA by inhalation at 10 ppm for 6 h; however, the results showed no subsequent increase in blood FA concentrations [12].

Regardless of these findings, many investigators are conducting studies to identify biomarkers of FA genotoxicity and cytotoxicity in other tissues. In Costa’s study, the frequency of sister chromatic exchanges in peripheral blood lymphocytes were significantly increased in FA exposed workers [13]. A study conducted by Schlosser [14] showed that levels of cross-links were generally higher in workers exposed to FA for longer periods, suggesting that DNA–protein cross-links can be used as a marker for biological monitoring of FA exposure. In Zhang’s study [15], the leukemia-related chromosome changes of monosomy 7 and trisomy 8 were found in the peripheral blood lymphocytes of workers exposed to FA; however, other studies have reported conflicting results. In Kun Lu’s study [16], exogenous FA DNA-adducts were only found in the nasal passages of rats exposed to FA, which supports a cytotoxic mechanism for carcinogenesis in the respiratory nasal epithelium, but does not address the mechanism for leukemia.

In recent decades, metabonomic approaches have been utilized when screening for metabolic products in biological fluids and tissues that might be useful for determining the toxicity of various compounds [17]. Metabolites are the intermediates and products of metabolism, and must be derived from the actions of proteins and the genes that code them [18]. In many cases, observed changes in metabolites can be related to a specific chemical exposure, clinical syndrome or disease [19]. Metabonomic approaches have been shown to be useful for monitoring exposure to environmental chemicals, determining preclinical toxicities, and identifying biomarkers of disease, and are applicable for use with samples obtained by non-invasion methods, such as urine samples [20,21].

In this study, we sought to determine whether the profile of endogenous metabolites in urine changed following exposure to FA, even though previous studies could not detect significant changes in the levels of FA in blood and urine. Assays for immune toxicity were used to determine whether a toxic event had been induced, liquid chromatography coupled with time of flight-mass spectrometry (LC-TOF-MS) was applied to examine variations of small molecule metabolites, and principal component analysis (PCA) was used to identify potential biomarkers.

## 2. Results and Discussion

### 2.1. Effects of FA on Immune Organs and Function

**Figure 1:**
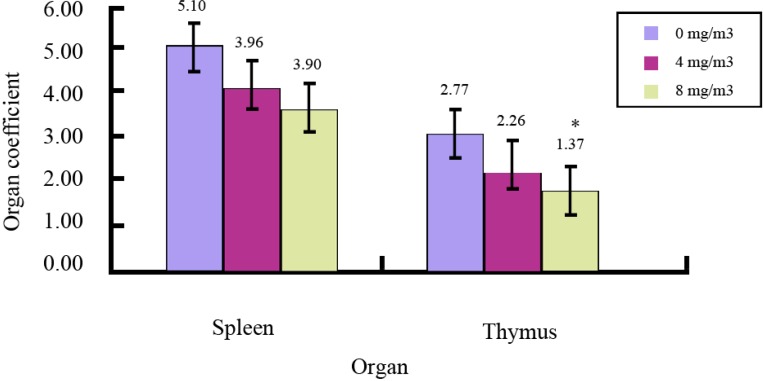
Immune organ coefficients of formaldehyde (FA) exposed mice compared with control mice. * Significant difference compared with control group (*p* < 0.05).

Immune cells undergo maturation in the spleen and thymus, and we therefore examined the spleen and thymus weights of mice in the three experimental groups; the results are shown in Figure 1. There were significant decreases in the spleen and thymus weights of FA exposed mice compared to the corresponding weights in control mice. There was also a significant decrease in T lymphocyte proliferation in the FA exposed groups (Table 1). These findings agreed with those in previous reports which stated that FA produced significant decreases in T-cell numbers and lymphocyte proliferation both *in vivo* and *in vitro* [7,8,22]. Additionally, previous studies reported that FA metabolism capable of generating ROS [17] was observed in the bone marrow, peripheral blood mononuclear cells, liver and spleen [23,24].

**Table 1:** T lymphocyte proliferation rates in spleen cells of FA exposed mice.

Dose (mg/m^3^)	Absorbance (A)	Proliferation Rate
0	0.485 ± 0.022	1
4	0.235 ± 0.038 *	0.485
8	0.208 ± 0.032 *	0.429

* Significant difference compared with control group (*p* < 0.05).

In our current study, the residual levels of ROS and SOD activity were examined, and the results are shown in Table 2. ROS levels were significantly increased in the spleen cells obtained from mice exposed to 8 mg/m^3^ FA, and SOD activity was significantly decreased in both the 4 and 8 mg/m^3^ exposure groups. Based on these adverse effects on the immune organs and cells, we believe that exposure to FA by inhalation caused significant immune toxicity in this study.

**Table 2:** ROS level and SOD activity in spleen cells from FA exposed mice.

Dose (mg/m^3^)	ROS Level (MFI)	SOD (U/mg prot)
0	2718.60 ± 355.47	10.80 ± 3.22
4	2988.67 ± 394.04	5.33 ± 1.06 *
8	3683.50 ± 332.06 *	2.26 ± 0.61 **

* Significant difference compared with control group (*p* < 0.05); ** Significant difference compared with 4 mg/m3 group (*p* < 0.05).

We originally sought to determine whether exposure to inhaled FA could produce biochemical changes, even if FA could not be detected in the blood and urine. We, therefore, utilized LC-TOF-MS to test for variations in the biochemical composition of urine following exposure to FA.

### 2.2. LC/MS Fingerprinting of Urine from Mice Exposed to FA, and Principal Component Analysis

**Figure 2:**
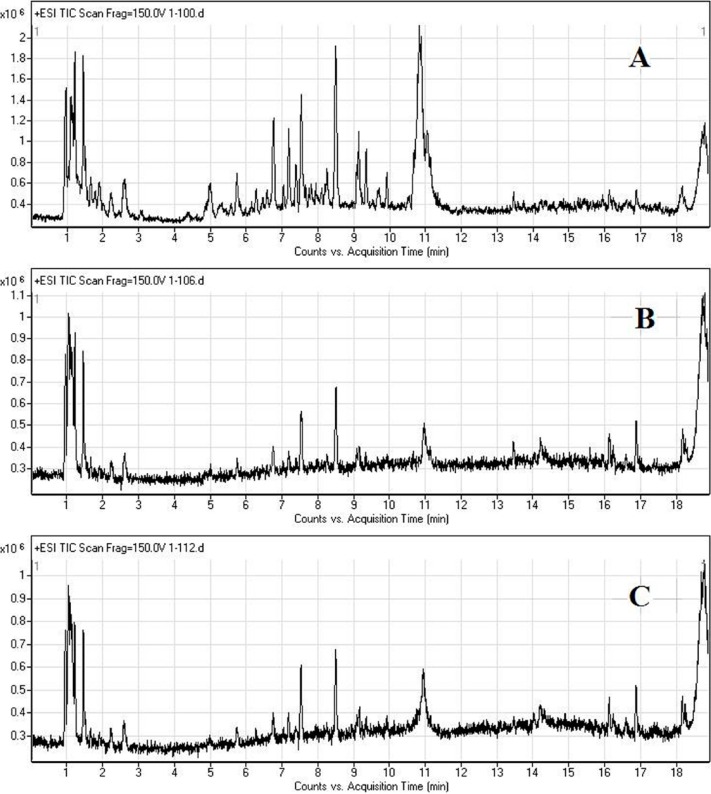
Total ion currents of mouse urine samples obtained from control group (**A**); FA 4 mg/m^3^ group (**B**); and FA 8 mg/m^3^ group (**C**) after 7 days of FA exposure as determined using LC/MS (positive mode).

Full-scan detection of quantitative information for urine metabonomics was obtained at the positive ion mode. As shown in Figure 2, there were significant differences in the total ion currents, especially from 4–12 min, obtained from the urine of mice in the control and FA exposed groups. These results suggest that certain endogenous metabolites may have been altered by FA exposure.

**Figure 3:**
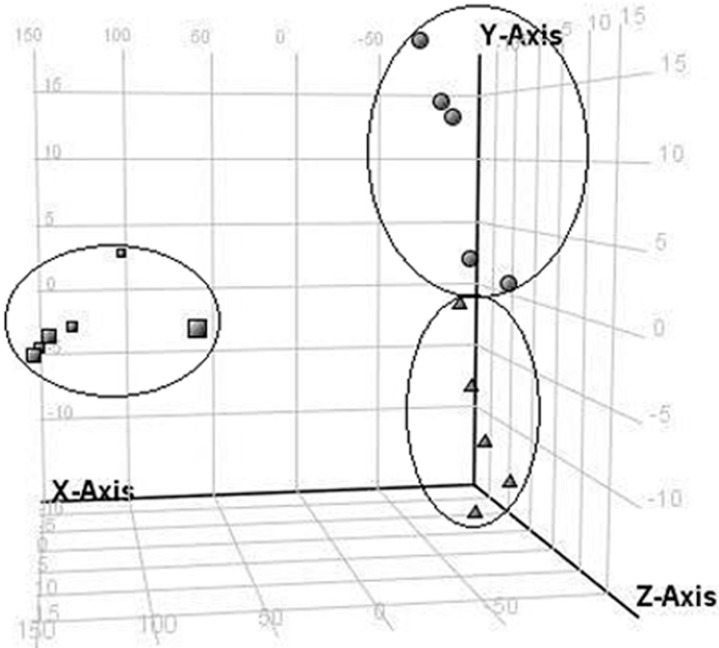
PCA score plot resulting from urine metabolic profiling of control (■), FA 4mg/m^3^ group (●), and FA 8 mg/m^3^ group (▲) on day 7.

Principal component analysis (PCA) is useful for distinguishing the amounts of biochemical endpoints retrieved from individual samples. Changes in metabolites were analyzed using one-way ANOVA (*p* < 0.05, fold-change ≥ 2), and PCA was used to select potential biomarker candidates that could differentiate between the control and FA exposed groups. A PCA score plot based on the urine metabolic profiles at different dose-points is shown in Figure 3. The urine samples tended to cluster at different locations at different doses, and the assembly of samples showed a unique metabolic pattern at each dose. In total, the results indicated that urine metabolic patterns significantly changed following FA inhalation.

### 2.3. Discrimination of Changed Endogenous Metabolites

Twelve common metabolites which showed significant alterations at two intervals were selected to undergo further analysis with LC-TOF-MS. For further identification, additional databases were searched according to exact mass and fragment ions as described in the methods section. Finally, two potential biomarker candidates for FA exposure (hippuric acid and cinnamoylglycine) were identified (Table 3), and found to be significantly down-regulated in the urine of FA exposed mice. In the present study, exposure to FA at concentrations of 4 and 8 mg/m^3^ produced 0.49- and 0.07-fold changes, respectively, in the hippuric concentrations of urine. However, the same FA exposures failed to produce dose-dependent changes in concentrations of cinnamoylglycine (0.34- and 0.37-fold changes at FA exposures of 4 and 8 mg/m^3^ respectively).

Hippuric acid has been used as a biomarker for exposure to toluene, and is formed by coenzyme A catalyzed conjugation of benzoic acid with glycine [25]. Additionally, hippuric acid is a component of non-protein nitrogen containing metabolites which result from protein and nucleic acid metabolism, and are found in urine [26]. Accordingly, down-regulation of hippuric acid levels has been reported in cases of renal disease [27,28]. In the present study, urinary levels of hippuric acid were significantly decreased in the FA inhalation group. This result suggests a potential toxicity related to down-regulation of hippuric acid; however the mechanism for such toxicity is unclear. Further investigations into this mechanism might focus on the liver and kidney toxicities induced by FA.

**Table 3:** Identification of significantly different metabolites in the urine of mice by LC-TOF-MS analysis.

No.	M + H	Retention Time (min)	Changes in FA Groups	MS/MS Fragmentation (20V)	Metabolites
1	180.0655	6.7560	down	(10V) 77.0389, 105.0336, 106.0372	Hippuric acid
2	206.0845	1.6843	down	103.0539, 104.0572, 131.0485, 132.0515	Cinnamoylglycine
3	313.1387	1.4592	down	85.0284, 120.0439, 189.9947, 190.9969, 191.9917	unknown
4	396.1673	8.5375	down	97.1007, 115.0574, 125.0948, 203.1085, 397.1463	unknown
5	467.1609	7.0492	down	261.1174, 305.1073, 306.1103, 467.1604, 468.162	unknown
6	202.1184	1.2264	down	60.0812, 70.0658, 71.0599, 85.0289, 100.0757	unknown
7	461.2593	7.1166	down	86.0953, 102.0535, 183.1111, 217.0805, 243.1319	unknown
8	473.2956	8.5641	down	112.0533, 201.0343, 203.0139, 215.998, 257.1149	unknown
9	105.0369	6.7562	down	(low abundance)	unknown
10	326.1070	1.7726	down	149.0443, 166.0714, 194.0662, 195.068	unknown
11	204.1244	1.2209	down	57.036, 58.0683, 60.0835, 71.0605, 85.0308	unknown
12	568.27	1.4622	down	(low abundance)	unknown

Cinnamoylglycine might be regulated by PPAR-α, a member of the nuclear receptor super family that functions in lipid metabolism. This is suggested by the finding that levels of cinnamoylglycine were increased nine fold in the urine of mice treated with the PPAR-α activator Wy-14,643 [29]. Cinnamoylglycine has also been found concordantly and significantly altered in the tissues and serum of mice with kidney cancer [30]. FA might induce down-regulation of cinnamoylglycine levels through PPAR-α inhibition. Further investigations into the mechanism of cinnamoylglycine regulation may provide new clues for understanding the toxic mechanism of FA.

## 3. Experimental Section

### 3.1. Animals and FA Exposure

All animals’ experiments were carried out in strict accordance with the recommendations of the Guide for the Care and Use of Laboratory Animals of the State Committee of Science and Technology of the People’s Republic of China. The protocol of experiments was reviewed and approved by the Research Ethics Committee of the Southeast University (approval number: 20,120,036).

Eighteen Balb/C mice (nine males and nine females, weighing 18–22 g) were obtained from the laboratory animal center of Nanjing Medical University (Nanjing, China, License Number: SCXK (su) 2002-0031). The mice were acclimatized for 1 week in a specific-pathogen free animal facility prior to administration of substances. The animals were maintained under a 12-h light/12-h dark cycle at a temperature of 25 ± 2 °C, and relative humidity of 45%–65%.

Three male mice and three female mice were randomly assigned to one of three groups consisting of two FA (AR, Xilong Chemical Co., Ltd., Shantou, China) exposure groups (4 and 8 mg/m^3^) and a non-exposed control group. Mice in the exposure groups were exposed to FA for 6 h per day for 7 consecutive days in an 8050 G-inhalation exposure chamber (Hepu, Co., Ltd., Tianjin, China). During the inhalation exposure, air samples were collected from the chamber, and FA concentrations were monitored. The chamber parameters used during exposure were temperature, 22 ± 1 °C; humidity, 45%; gas flow rate, 3 L/min. Urine samples were collected on the 7th day of exposure, and the mice were then sacrificed. The spleen and thymus of each mouse were excised and weighed, and the relative organ weights were calculated as the ratio of the organ weight and body weight.

### 3.2. FA Induced Immune Toxicity to Spleen Cells

Lymphocyte proliferation studies were conducted as follows. The spleen cells of each mouse were isolated respectively, plated at a density of 10^5^ cells/well, and co-cultured with ConA (5 μg/mL) in 96-well plates. The cells were cultured for 48 h at 37 °C in an atmosphere of 5% CO_2_, and then centrifuged at 1000× *g* for 10 min. Lymphocyte proliferation was examined using the 3-(4,5-dimethyl-2-thiazolyl)-2,5-diphenyl-2-*H*-tetrazolium bromide method. ROS levels and SOD activity in spleen cells were detected with a reactive oxygen species assay kit (Nanjing Keygen Biotech. Co., Ltd., Nanjing, China) and a superoxide dismutase assay kit, respectively (Nanjing Jian Cheng Bioengineering Institute, Nanjing, China).

The oxygen species assay was based on the ROS dependent fluorescence intensity (MFI) as detected by a fluorescent probe containing 2',7'-dichlorofluorescin diacetate. SOD activity was defined as the reduction rate of superoxide anions determined by a colorimetric method.

### 3.3. Sample Preparation and HPLC/MS Analysis

Non-targeted analyses were used to identify altered patterns of metabolites in urine in an Agilent 6224 TOF LC-MS system (Agilent), and the molecules obtained from the non-targeted analyses were identified by LC/6530 Q-TOF-MS (Agilent, Santa Clara, CA, USA). Urine samples were thawed at room temperature and centrifuged at 13,000× *g* for 15 min at 4 °C. Then, 300 μL of methanol was added to a 100-μL aliquot of urine, and the solution was vortex mixed to precipitate proteins. The supernatants were then centrifuged at 13,000× *g* for 15 min at 4 °C and transferred to auto-sampler vials. For LC-MS detection, a 1-μL aliquot of each sample was injected onto a ZORBAX Eclipse Plus C18 column (3.00 mm × 100 mm × 1.8 μm, Agilent) housed in TOF LC-MS system. The mobile phases were 0.1% formic acid in water (A) and 0.1% formic acid in acetonitrile (B), and the flow rate was 0.4 mL/min. The column and auto sampler were maintained at 35 and 4 °C, respectively, and the positive ion mode was set for the mass detection. The source parameters used were a drying gas flow rate of 9 L/min, gas temperature of 350 °C, nebulizer gas pressure of 40 psig, Vcap of 4000 V, fragmentor of 150 V, skimmer of 60 V, and scan range of *m*/*z* 50–1000. The tuning calibration solution (Agilent) was used as the lock mass (*m*/*z* = 121.050873, 922.009798) at a flow rate of 30 μL/min, via a lock spray interface for accurate mass measurement.

The metabolites obtained from non-targeted analyses were identified by MS/MS analysis conducted in a targeted MS/MS mode with collision energies of 10, 20, and 40 V, and a scan rate of 1 (MS/MS) scans/s.

### 3.4. Processing of Metabolite Data

Data obtained from LC-MS studies were analyzed using Masshunter Data Analysis Software (Ver B.02.01, Agilent Technologies, Barcelona, Spain). The molecular features of various samples were analyzed using Masshunter Qualitative Analysis Software (Agilent Technologies), and methods based on the Molecular Feature Extractor (MFE) algorithm. Finally, Masshunter Mass Profiler Professional Software (Ver B.02.02, Agilent Technologies) was used to perform non-targeted analyses of extracted features, which showed a minimum absolute abundance of 2000 counts and a minimum of two ions. Data from PCA analysis were used to identify several distinct variables that might serve as potential biomarkers. Compounds from different samples were aligned using an RT window of 0.2% ± 0.15 min and a mass window of 10 ppm ± 2.0 mDa. To correct for individual bias, only common features found in at least 75% of samples under the same conditions were analyzed. The metabolites with exact masses were searched in various databases (METLIN, HMDB, LIPID MAPS and KEGG).

### 3.5. Statistics

The methods used for non-targeted metabonomic analyses are described in the data processing section of this paper. Other statistical analyses were performed using SPSS 15.0 software (SPSS, Chicago, IL, USA). Multiple comparisons were analyzed using one-way ANOVA, and *p*-values <0.05 were regarded as statistically significant.

## 4. Conclusions

Small molecule metabolite biomarker candidates found in the urine of Balb/c mice exposed to inhaled FA were investigated using an LC-MS-based metabonomics approach. Significant decreases in T lymphocyte proliferation rates in the spleen and thymus were found to characterize the immune toxicity induced by FA. Additionally, increased ROS levels accompanied by decreased SOD levels in the spleen support the presence of injury to the immune system. Levels of 12 small molecule metabolites were found to be altered in the urine of mice exposed to FA, and PCA analysis showed that urine from the control and FA exposed mice could be discriminated based on this information. Hippuric acid and cinnamoylglycine with two interval changes were identified using exact mass and fragment ions. The molecular mechanisms of how FA induced the decreased urine levels of hippuric acid and cinnamoylglycine are still unknown and still need to be resolved. However, several studies have suggested that hippuric acid levels are altered in the presence of abnormal liver and kidney function, and cinnamoylglycine is a biomarker for activation of PPAR-α and altered in kidney cancer. The decreased levels of hippuric acid and cinnamoylglycine in urine might serve as biomarker candidates for FA exposure. Actually, hippuric acid and cinnamoylglycine are biomarkers for an adverse effect of FA and appropriate controls for other compounds that have a similar effect have to be performed to exclude false positive results. In conclusion, our study identified potential new biomarker candidates for FA exposure, and such markers may assist in understanding how exposure to high levels of FA can result in serious injuries, even when excess FA cannot be detected in blood and urine.
